# Probiotics and Atopic Dermatitis in Children

**DOI:** 10.3390/ph5070727

**Published:** 2012-07-06

**Authors:** Fabio Meneghin, Valentina Fabiano, Chiara Mameli, Gian Vincenzo Zuccotti

**Affiliations:** Department of Pediatrics, Luigi Sacco Hospital, Università degli Studi di Milano, Via GB Grassi 74, 20157 Milan, Italy; Email: fabiano.valentina@hsacco.it (V.F.); mameli.chiara@hsacco.it (C.M.); gianvincenzo.zuccotti@unimi.it (G.V.Z.)

**Keywords:** probiotics, atopic dermatitis, prevention, treatment

## Abstract

There is increasing interest in the potential beneficial role of probiotic supplementation in the prevention and treatment of atopic diseases in children. Probiotics are defined as ingested live microorganisms that, when administered in an adequate amount, confer a health benefit to the host. They are mainly represented by Lactobacilli and Bifidobacteria. Several epidemiological data demonstrate that intestinal microflora of atopic children is different from the one of healthy children. Many literature data show that probiotics may modulate the intestinal microflora composition and may have immunomodulatory effect. Based on this hypothesis, probiotics are supposed to confer benefits to allergic diseases. Administration of probiotics when a natural population of indigenous intestinal bacteria is still developing could theoretically influence immune development by favoring the balance between Th1 and Th2 inflammatory responses. For this reason, some studies have evaluated the potential impact of probiotics supplementation in the prevention of atopic dermatitis, with contrasting results. Clinical improvement in immunoglobulin (Ig)E-sensitized (atopic) eczema following probiotic supplementation has been reported in some published studies and the therapeutic effects of probiotics on atopic dermatitis seemed to be encouraging. However, as far as the usefulness of probiotics as a prevention strategy is concerned, results are still inconclusive. In fact, the clinical benefits of probiotic therapy depend upon numerous factors, such as the type of bacteria, dosing regimen, delivery method and other underlying host factors, such as age and diet. More studies are still needed to definitively prove the role of probiotics in the treatment of allergic eczema.

## 1. Introduction

In 1908, the Russian immunologist Elie Metchnikoff suggested that lactic acid bacteria in fermented milk may confer health benefits to humans [1] and paved the way for future research on the potential beneficial effects of probiotics. Since then, more than a century has passed and probiotics continue to capture the attention of the scientific community. The Food and Drug Organization of the United Nations (FAO) and World Health Organization (WHO) have defined probiotics as “live microorganisms which when administered in adequate amounts confer a health benefit to the host” [2]. A number of probiotics are currently commercially available; however, not all bacterial strains can be considered probiotics. Probiotics should in fact have some fundamental characteristics: they should be of human origin, non-pathogenic in nature and resistant to destruction by technical processing and by bile and gastric acids, they should be able to adhere to intestinal mucosa and colonize it, produce antimicrobial substances, modulate immune response and influence human metabolic activities [3]. The two most important bacterial genera with probiotic properties are *Lactobacillus* and *Bifidobacterium* [4]. Numerous studies evaluated the potential activities of probiotics in the prevention and treatment of human diseases, including allergic diseases and atopic dermatitis in particular.

Atopic dermatitis (AD) is a common childhood-onset skin disease which, in nearly half of the cases, has an allergic origin. It is clinically characterized by a chronic or relapsing course, with periods of disease exacerbations alternating with various degrees of remission. The skin is dry and pruritus represents the hallmark symptom; it is disturbing and causes skin excoriations, substantially affecting the quality of life of patients and their relatives [5]. The precise etiology, pathophysiology, and pathogenesis of AD are not yet fully understood. Nevertheless, it can be considered the result of a complex interaction between genetic [6] and environmental factors, which are both implicated in the predisposition and development of disease [7]. The disease is associated with abnormalities in skin barrier molecules and in cells of the inflammatory response. The immunoregulatory abnormalities are characterized by impaired Th1-Th2 cytokines balance, with a prevalent activation of Th2 cytokines, which results in increased serum immunoglobulin IgE and elevated total circulating eosinophil count [8].

## 2. Hypothesized Probiotics Mechanisms of Action in Allergic Diseases

As generally observed for other allergic diseases, incidence of AD has shown a twofold to threefold increase in the last decades, and currently it is estimated to be nearly 20% in the industrialized countries [9]. The first hypothesis proposed to explain the phenomenon of the increasing incidence of atopic diseases is the so-called “hygiene hypothesis” [10]. Immune response in neonates is dominated by Th2 cytokines, but, during the first year of life, immune responses shift to the Th1-based ones as a result of the repeated exposure to different infectious antigens [11]. The hygiene hypothesis is based on the finding that the prevalence of allergic diseases is inversely related to high-standard hygienic and sanitary conditions: smaller family sizes, less crowded accommodations, routine vaccinations and widespread use of antibiotics have all contributed to reduce childhood contact with pathogens, decreasing the Th1-driven immune response and, on the contrary, favoring the persistence of the neonatal Th2-mediated one, which may later lead to appearance of allergic diseases [10].

More recently, another hypothesis has been proposed. The host’s primary microbial stimulation occurs with the establishment of the gut microflora, and exposure to commensal microflora or to specific bacterial strains may represent a key modulator of the immune system which may prevent development of atopic diseases [12,13]. At birth, the gastrointestinal tract of the newborn is sterile. After birth, neonatal microflora develops as a result of different intrinsic and extrinsic factors [14]. The developing microflora in the early postnatal period is involved in the activation of innate and adaptive immunity and the continuous microbial stimulus from the developing microflora is required for the successful maturation of the gut mucosal immune system. As a consequence, a lacking or inadequate microbial stimulus results in reduction of the intestinal surface area, alteration of mucosal enzyme patterns, mucosal barrier and mucosal IgA system [15,16], and abrogation of oral tolerance, that is the capacity of tolerating nonpathogenic antigens previously encountered on mucosal surfaces [17]. Unbalanced gut microflora favors the persistence of a neonatal Th2-oriented immune response, favoring development of atopy.

It has been observed that gut microflora of atopic children is characterized by a reduced neonatal bifidobacteria to clostridia ratio, caused by augmented clostridia and reduced bifidobacteria colonization [18,19]. If unbalanced microflora may favor the development of atopic diseases, probiotics may be helpful because of their capacity of balancing the gut microecology, restoring the normal intestinal permeability, improving immunological gut barrier function and downregulating the production of pro-inflammatory cytokines. Moreover, it has been suggested that beneficial effects of probiotics against atopic diseases are associated with stimulation of Toll-like receptors (TLRs). Intestinal epithelial cells produce various pattern-recognition receptors that recognize microbial antigens, also called pathogen-associated molecular patterns (PAMPs). A class of these receptors are the TLRs. To date, 11 mammalian TLRs have been identified, whose expression pattern is cell type specific. Stimulation of TLRs by various PAMPs induces the differentiation of T cells, with Th1 cells that mediates cellular responses and inflammatory reactions and Th2 cells which mediates humoral responses, antiparasitic defense and allergic reactions [20]. Microflora-host interaction mediated by TLRs may play a role in the susceptibility to food allergy as hypothesized in the study by Bashir and colleagues [21] in which two groups of mice with a mutation or a deficiency of TLR-4, in contrast to TLR-4 wild type mice, showed a typical IgE-mediated immune response when exposed to allergic antigens.

Given all the aforementioned evidences, numerous studies have evaluated the potential benefits of probiotics in children affected by allergic diseases, and in particular by AD, with contrasting results. We conducted a systematic review of the randomized controlled trials about the use of probiotics in prevention and treatment of pediatric atopic dermatitis (PAD), published in Pub-Med from 1997 to nowadays, in English language, using key words “probiotics”, “atopic dermatitis”. “children”, “prevention”, “treatment”. To our knowledge, this is the most up-to-date review on this topic.

## 3. Probiotics in the Prevention of Pediatric Atopic Dermatitis

Numerous studies have evaluated the potential efficacy of probiotics in the prevention and treatment of allergic diseases in general, and of AD in particular, showing conflicting results. To date, 17 trials of probiotics in the prevention of PAD have been published (Table 1). The majority of prevention studies performed from 2001 onwards focused on children at high risk for atopy, and in most of them, probiotics were administered 2–4 weeks prenatally to pregnant women and postnatally to infants for 6–12 months. The most frequently studied probiotic strain is *Lactobacillus rhamnosus GG* (LGG). Kalliomäki and colleagues [22] first evaluated efficacy of LGG in a double-blind randomized control trial (RCT) in a group of 132 infants at high risk for atopy. At the follow-up after two years, frequency of AD in infants supplemented with probiotics was half that of the placebo group (23% *vs*. 46%, respectively). The same research group subsequently evaluated the same cohort of infants four and seven years later confirming the persistence of a protective effect of probiotics against development of AD [23,24]. Preventive effects of LGG on AD were also demonstrated by Rautava *et al*. [25] in 2002, who evaluated its efficacy on 57 high-risk children breastfed by mothers supplemented with LGG strain. In the supplemented group the risk of developing atopic eczema was 15%, while in the placebo group it was 47% [relative risk (RR): 0.32 (95% confidence interval (CI), 0.12–0.85); *p* = 0.0098]. On the contrary, Kopp *et al*. [26] found no reduction of AD in high-risk infants supplemented with LGG. Supplementation with mixed probiotic strains was also evaluated. Kukkonen *et al*. [27] evaluated in a RCT the efficacy of supplementation with a mixture of 4 different probiotic bacterial strains in a large cohort of infants at high risk for atopy. At two years of age, probiotics did not reduce the cumulative incidence of all atopic diseases but significantly prevented both eczema and atopic eczema [Odds Ratio (OR) 0.74 (95% CI, 0.55–0.98); *p* = 0.035 for eczema; OR 0.66 (95% CI, 0.46–0.95); *p* = 0.025 for atopic eczema]. More recently, a trial by Dotterud *et al*. [28] studied a mixture of LGG, *L. acidophilus La-5, Bifidobacterium animalis* subsp. *lactis* administered prenatally to a population of non-selected mothers from the 36th week of gestation and postnatally to their exclusively breastfed infants for the first 3 months. They showed reduced cumulative incidence of AD after supplementation with mixed probiotics: in the intention-to-treat (ITT) analysis the OR was 0.51 in infants supplemented with probiotics respect to placebo group (95% CI, 0.30–0.87; *p* = 0.013). In other three RCTs evaluating the efficacy of mixed probiotic strains, risk of sensitization and incidence of AD were reduced after probiotics supplementation [29,30,31]. On the contrary, no preventive effects of mixed probiotic strains on incidence of PAD were observed in other two RCTs [32,33]. A significant reduction of atopic eczema was demonstrated only in cesarean-delivered children supplemented with probiotics respect to placebo group (24.3% *vs*. 40.5%; OR, 0.47; 95% CI, 0.23% to 0.96%; *p* = 0.035); whereas frequency of eczema (39.3% *vs*. 43.3%) and atopic eczema (24.0% *vs*. 25.1%) did not differ between groups [33]. A RCT published in 2008 [34] compared the efficacy of two different bacterial strains, *L. rhamnosus* and *Bifidobacterium animalis* subsp. *lactis*, in the prevention of pediatric eczema. At two years of age, only *L. rhamnosus* showed efficacy in reducing cumulative prevalence of eczema, whereas no effects were demonstrated for *B. animalis*. Supplementation during weaning with *Lactobacillus F19* reduced cumulative incidence of atopic eczema in 179 infants in one trial [35]. On the contrary, two trials [36,37] with *Lactobacillus acidophilus* and *L. reuteri* ATCC, respectively, showed no preventive effects of probiotics on incidence of AD. At six months, there was no difference in rates of AD between the supplemented group and the placebo group (25.8% *vs*. 22.7%, respectively; *p* = 0.629) in the first study [36]; in the second trial, cumulative incidence of atopic eczema was similar in the treated and untreated group (36% *vs*. 34%) [37]. Prenatal supplementation of pregnant women did not result in reduction of AD incidence in infants: incidence of AD and IgE-associated eczema in 1-yaer old infants at high risk for atopic diseases, born to 250 mothers supplemented with LGG from the 36th week of gestation to delivery, did not show a significant reduction respect to mothers who have not been supplemented [38]. Some recently published reviews and meta-analysis, including a Cochrane Collaboration review, concluded that there is still insufficient evidence for supporting the use of probiotics in the prevention of AD [39,40,41,42], whereas some others, including the most recently published one, stated that convincing evidence exists for encouraging supplementation with probiotics for the prevention of AD in children [43,44,45,46].

## 4. Probiotics in the Treatment of Pediatric Atopic Dermatitis

In 1997, for the first time, a Finnish group of authors showed a significant improvement in atopic dermatitis children after a supplementation with probiotics. From then on, 19 trials regarding the treatment of PAD with probiotics have been published (Table 2) and even some meta-analysis and reviews, but current results and conclusions are not very convincing.

The majority of studies investigated the effect of one probiotic strain *vs.* placebo in PAD. Majamaa and Isolauri [47] studied a group of infants with atopic eczema and a history of cow’s milk allergy. Patients were put on a cow’s milk elimination diet and randomized to receive extensively hydrolyzed whey formula with or without *LGG*. Moreover, in 11-breastfed atopic infants, LGG supplementation was given to nursing mothers. Results of this trial showed a significant improvement of SCORAD score after 1 month of supplementation only in the probiotic group. Three years later, the same group of Isolauri *et al*. [48] randomized 27 fully breastfed atopic infants to receive for 4 weeks an extensively hydrolyzed whey formula with *LGG* or *Bifidobacterium lactis Bb-12* or placebo. An evaluation two months later demonstrated a significant improvement in SCORAD score in probiotic groups. The group of Kirjavainen performed 2 trials in 2002 [49] and 2003 [50] in which infants with early onset atopic eczema were randomized to receive in the first study a formula contained *Bifidobacterium lactis Bb-12 vs.* placebo, and in the second one a formula with viable or heat inactivated *LGG vs.* placebo. Results showed that probiotics supplementation modulates the composition of gut microbiota in a manner that may alleviate allergic inflammation and only viable probiotic strains are safe and potentially helpful for PAD treatment. In 2005 Weston *et al*. [51] recruited 53 children with moderate to severe AD to evaluate the effect of *Lactobacillus fermentum*. Results showed that an 8-week supplementation was beneficial in improving extent and severity of AD. More recently Woo *et al*. [52] demonstrated that a 12-week supplementation of *Lactobacillus Sakei* in 75 children with atopic eczema-dermatitis syndrome was associated with a significant clinical improvement and a decrease of chemokine levels.

On the contrary, other trials focused on a single strain supplementation, did not confirm the positive effects of probiotics in AD treatment. Brouwer *et al.* [53] randomized 50 infants with AD to receive *Lactobacillus rhamnosus* or *Lactobacillus GG* or placebo in a hydrolyzed whey formula for 3 months.

**Table 1:** Probiotics in the prevention of Atopic Dermatitis (AD) in children.

Study	Partecipants	Probiotics	Type of study	Duration	Outcome
Kalliomäki *et al*. [22]	132 high-risk infants for atopy	*Lactobacillus rhamnosus* GG (1 × 10^10^ cfu daily)	R, DB, PC	From 2–4 weeks prenatally to 24 weeks postnatally	At 2 years AD frequency halved in probiotic group *vs*. placebo (23% *vs*. 46%)
Kalliomäki *et al*. [23]	107 high-risk infants for atopy	*Lactobacillus* *rhamnosus* GG	Same cohort of 2001	Same cohort of 2001	4 years follow-up: protective effect against AD still detected in probiotic group
Kalliomäki *et al*. [24]	116 high-risk infants for atopy	*Lactobacillus* *rhamnosus* GG	Same cohort of 2001	Same cohort of 2001	7 years follow-up: incidence AD decreased during this period
Rautava *et al*. [25]	57 high-risk infants for atopy	*Lactobacillus rhamnosus* GG (2 × 10^10^ cfu daily)	R, DB, PC	Prenatal and 3 months postnatally to breastfeeding mothers	Reduction in AD prevalence in infants brestfed by mothers in probiotic group *vs*. placebo
Kopp *et al*. [26]	94 high-risk infants for atopy	*Lactobacillus* *rhamnosus* GG (5 × 10^9^ cfu twice daily)	R, DB, PC	4–6 weeks prenatally and 6 months after delivery	No reduced incidence of AD and no modification of severity
Kukkonen *et al*. [27]	925 high-risk infants for atopy	*Lactobacillus* *rhamnosus* GG (5 × 10^9^ cfu), *L. rhamnosus* LC705 (5 × 10^9^ cfu), *Bifidobacterium breve* Bb99 (2 × 10^8^ cfu), *Propionibacterium* *freudenreichii* ssp. Shermanii JS (2 × 10^9^ cfu) twice daily	R, DB, PC	From 2–4 weeks prenatally to 6 months post delivery	Probiotics reduced AD
Dotterud *et al*. [28]	292 infants without risk factor for atopy	*Lactobacillus rhamnosus* GG (5 × 10^9^ cfu), *L. acidophilus* La-5 (5 × 10^9^ cfu), *Bifidobacterium animalis* subsp. *lactis* (5 × 10^9^ cfu) daily	R, DB, PC	To non-selected (for atopy) mothers from 36 weeks of gestation to 3 months postnatally (all infants breastfed)	Probiotics reduced the cumulative incidence of AD among children at 2 years of age
Huurre *et al*. [29]	140 high-risk infants for atopy	*Lactobacillus rhamnosus* GG (1 × 10^10^ cfu) and *Bifidobacterium lactis* (1 × 10^10^ cfu) daily	R, DB, PC	To mothers from the 1st trimester of pregnancy to the end of exclusive breastfeeding	Risk of sensitization at 12 months of age for infants of atopic mothers, was reduced by use of probiotics during pregnancy and lactation
Niers *et al*. [30]	102 high-risk infants for atopy	*Bifidobacterium Bifidum* (1 × 10^9^ cfu), *Bifidobacterium lactis* (1 × 10^9^ cfu), *Lactococcus* lactis (1 × 10^9^ cfu) daily	R, DB, PC	To mothers from 6 weeks prenatally and to infants for 12 months postnatally	Probiotics shows a preventive effect of incidence of AD in high-risk infants during the first 2 years of life
Kim *et al*. [31]	112 high-risk infants for atopy	*Bifidobacterium Bifidum* (1.6 × 10^9^ cfu), *Bifidobacterium lactis* (1.6 × 10^9^ cfu), *Lactobacillus Acidophilus* (1.6 × 10^9^ cfu) daily	R, DB, PC	To mothers from 8 weeks prenatally and to mothers/infants for 6 months postnatally	At 6 and 12 months the prevalence of AD in probiotics group was reduced than in placebo group. Cumulative incidence of AD significantly lower in probiotics group at 12 months
Soh *et al*. [32]	253 high-risk infants for atopy	*Bifidobacterium longum* (1 × 10^7^ cfu), *Lactobacillus rhamnosus* (2 × 10^7^ cfu) daily	R, DB, PC	To infants from birth to 6 months with milk formula	No effect on prevention of AD in the first year of life
Kuitunen *et al*. [33]	891 high-risk infants for atopy	*Lactobacillus rhamnosus* GG (5 × 10^9^ cfu), *L. rhamnosus* LC705 (5 × 10^9^ cfu), *Bifidobacterium breve* Bb99 (2 × 10^8^ cfu), *Propionibacterium freudenreichii* ssp. shermanii JS (2 × 10^9^ cfu) twice daily	R, DB, PC	To mothers from 36 weeks of gestation prenatally and to infants postnatally until 6 months of age	At 5 years of age no significant difference appeared in frequencies of AD and IgE associated (atopic) eczema between probiotics and placebo groups. Only cesarean-delivered children had significantly fewer incidence of AD
Wickens *et al*. [34]	474 high-risk infants for atopy	*L. rhamnosus* (6 × 10^9^ cfu) or *Bifidobacterium* animalis subsp. lactis (9 × 10^9^ cfu) daily	R, DB, PC	To mothers from 35 weeks prenatally to 6 months if breastfeeding and to infants from birth to 2 years	Only *L. rhamnsosus* reduced cumulative prevalence of AD by 2 years. No effects for *B. animalis*
West *et al*. [35]	179 infants	*Lactobacillus* F19 (1 × 10^8^ cfu daily)	R, DB, PC	To infants during weaning from 4 to 13 months of age	Cumulative incidence of AD at 13 months of life was lower in probiotic group
Taylor *et al*. [36]	178 high-risk infants for atopy	*Lactobacillus acidophilus* (3 × 10^9^ cfu daily)	R, DB, PC	Newborn of allergic mothers received probiotic or placebo for 6 months	At 6 months or 1 year no reduction in AD in probiotic group *vs*. placebo
Abrahamsson *et al*. [37]	188 high-risk infants for atopy	*Lactobacillus reuteri* ATCC (1 × 10^8^ cfu daily)	R, DB, PC	Prenatally to mothers from 36 gestational age and postnatally to infants for 1 year	For AD similar cumulative incidence for probiotic and placebo groups. In probiotic group less IgE-associated eczema during 2 years
Boyle *et al*. [38]	250 pregnant women carrying infants at high risk of atopy	*Lactobacillus rhamnosus* GG (1.8 × 10^10^ cfu) daily	R, PC	To pregnant women from the 36th week of gestation until delivery	At 1 year of age, no reduction in incidence of AD and IgE-associated eczema in infants of supplemented mothers

R: randomized; DB: double blind; PC: placebo-controlled.

**Table 2:** Probiotics in the treatment of Atopic Dermatitis (AD) in children.

Study	Partecipants	Probiotics	Type of study	Duration	Outcome
Majamaa and Isolauri [47]	27 infants (1st study) and 11 breastfed infants (2nd study)	*Lactobacillus rhamnosus* GG (5 × 10^8^ cfu/gm formula or 2 × 10^10^ cfu twice daily to nursing mothers)	R, DB, PC	1st: Cow’s milk elimination diet plus formula milk with or without LGG for 4 weeks. 2nd: LGG to mothers during breastfeeding for 4 weeks	After 4 weeks significant SCORAD score reduction (extent, intensity and subjective score) in probiotic group. No difference between groups at 2 months
Isolauri *et al*. [48]	27 infants with AD during breastfeeding	*Lactobacillus rhamnosus* GG (3 × 10^8^ cfu/g) or *Bifidobacterium lactis* Bb-12 (1 × 10^9^ cfu/g)	R, DB, PC	4 weeks	At 2 months, significant improve in SCORAD score in probiotics groups
Kirjavainen *et al*. [49]	21 infants with early onset AD assumed hydrolyzed whey formula	*Bifidobacterium lactis* Bb-12 (1 × 10^9^ cfu/g)	R, DB, PC	Not specified	Probiotic supplementation modulates composition of gut microbiota and alleviate symptoms of atopy
Kirjavainen *et al*. [50]	35 infants (5.5 months mean age) with AD and suspected (CMA)	Viable *Lactobacillus* GG (1 × 10^9^ cfu/g) or heat-inactivated LGG	R, DB, PC	Mean length of intervention period of 7.5 weeks	Significant reduction of SCORAD score in viable LGG group
Weston *et al*. [51]	53 children (6–18 months af age) with AD	*Lactobacillus fermentum* (1 × 10^9^ cfu) twice daily	R, DB, PC	8 weeks	Probiotic improved severity of AD in children
Woo *et al*. [52]	75 children (2–10 years of age) with AEDS	*Lactobacillus sakei* (5 × 10^9^ cfu) twice daily	R, DB, PC	12 weeks	Probiotic supplementation improved clinical severity of AEDS and decreased chemokine levels
Brouwer *et al*. [53]	50 children (<5 months of age) with AD and suspected CMA	*Lactobacillus rhamnosus* (3 × 10^8^ cfu/g) or *Lactobacillus* GG (3 × 10^8^ cfu/g)	R, DB, PC	12 weeks	No clinical or immunological effect of probiotics in children with AD
Folster-Holst *et al*. [54]	54 children (1–55 months of age) with moderate or severe AD	*Lactobacillus rhamnosus* strain GG (5 × 10^9^ cfu) twice daily	R, DB, PC	8 weeks	No significant difference in clinical outcome by administration of probiotic
Gruber *et al*. [55]	102 infants (3–12 months of age) with mild to moderate AD	*Lactobacillus rhamnosus* strain GG (5 × 10^9^ cfu) twice daily	R, DB, PC	12 weeks	No therapeutic effect of probiotic in mild-to-moderate AD
Nermes *et al*. [56]	37 infants (6.5 months mean age) with AD	*Lactobacillus rhamnosus* strain GG (3 × 15^7^ cfu/g) daily	R, DB, PC	12 weeks	SCORAD indices decreased in both probiotic and placebo groups
Gore *et al*. [57]	208 children (3–6 months of age) with AD	*Lactobacillus paracasei* (10^10^ cfu) or *Bifidobacterium lactis* (10^10^ cfu) daily	R, DB, PC	12 weeks	No significant difference in SCORAD between all groups at any time-point up to age 3 year
Rosenfeldt *et al*. [58]	43 children with AD 1–13 years old	*Lactobacillus rhamnosus* (10^10^ cfu) and *L. reuteri* (10^10^ cfu) twice daily	R, DB, PC	6 weeks	Moderate improvement of clinical severity of AD with administration of probiotics *Lactobacillus* strains
Rosenfeldt *et al*. [59]	41 children (mean age 4 years) with moderate and severe atopic dermatitis	*Lactobacillus rhamnosus* (10^10^ cfu) and L reuteri DSM (10^10^ cfu) twice daily	R, DB, PC	6 weeks	Probiotic supplementation may stabilize the intestinal barrier function with an improvement of SCORAD score
Viljanen *et al*. [60]	230 children (mean age 6.4 months) with AD and suspected CMA	LGG (5 × 10^9^ cfu) or mixture: LGG (5 × 10^9^ cfu), *L. rhamnosus* LC705 (5 × 10^9^ cfu), *Bifidobacterium breve* (2 × 10^8^ cfu), *Propionibacterium freudenreichii* ssp. Shermanii JS (2 × 10^9^ cfu) twice daily	R, DB, PC	4 weeks	Reduction in SCORAD score only in IgE-associated AD in LGG group
Sistek *et al*. [61]	59 children (1–10 years of age) with AD	*Lactobacillus rhamnosus* (2 × 10^10^ cfu/g) and *Bifidobacterium lactis* (2 × 10^10^ cfu/g) daily	R, DB, PC	12 weeks and follow-up until 18 weeks	Probiotics improved AD only in food sensitized children
Gerasimov *et al*. [62]	90 children (1–3 years of age) with moderate to severe AD	*Lactobacillus acidophilus* (5 × 10^9^ cfu) and *Bifidobacterium lactis* (5 × 10^9^ cfu) twice daily	R, DB, PC	8 weeks	Probiotics improved significantly clinical severity of AD with a greater decrease in SCORAD score in children supplemented
Yeşilova *et al*. [63]	40 children (1–13 years of age) with moderate to severe AD	*B. bifidum* (2 × 109 cfu), *L. acidophilus* (2 × 109 cfu), *L. casei* (2 × 109 cfu), and *L. salivarius* (2 × 109 cfu) twice daily	R, DB, PC	8 weeks	Probiotic in AD patients effectively reduced the SCORAD index
Passeron *et al*. [64]	39 children (>2 years of age) with moderate and severe AD	*Lactobacillus rhamnosus* Lcr35 (1.2 × 10^9^ cfu) plus prebiotic preparation vs prebiotic alone three times a day	R, DB	12 weeks	Both synbiotic and prebiotic alone significantly improve the manifestations of AD
Van der Aa *et al*. [65]	82 infants (0–7 months af age) with AD exclusively formula-fed	*Bifidobacterium* breve (1.3 × 10^9^ cfu/100 mL) plus prebiotic mixture	R, DB, PC	12 weeks	Probiotic and prebiotic not have beneficial effect on AD severity in infants
Wu *et al*. [66]	54 children (2–14 years of age) with moderate to severe AD	*Lactobacillus salivarius* (2 × 10^9^ cfu) plus prebiotic *vs.* prebiotic alone	R, DB	8 weeks	In probiotic group was seen a significantly lower SCORAD score at 10 weeks

R: randomized; DB: double blind; PC: placebo-controlled; CMA :cow’s milk allergy; LGG: *Lactobacillus rhamnosus* GG.

Results from this study showed no significant effect of probiotics on SCORAD score, sensitization, inflammatory parameters or cytokine production. In the same year another group of authors [54] confirmed the ineffective treatment with respect to clinical symptoms, use of topical corticosteroids and antihistamines, immunological parameters or health-related quality of life, of *Lactobacillus rhamnosus strain GG* in 54 infants with moderate to severe AD. In 2007, Gruber *et al*. [55] performed a large trial with 102 infants with mild-to-moderate AD, who were randomized to receive a supplementation with *Lactobacillus rhamnosus GG* or placebo for 12 weeks, finding no therapeutic effect. Nermes *et al*. [56], in a smaller group of atopic infants randomized to receive *Lactobacillus rhamnosus GG* or placebo for 3 months demonstrated the same clinical improvement in SCORAD indices in treated and untreated groups. Very recently Gore *et al*. [57] investigated a dietary supplementation of 208 atopic infants with *Lactobacillus paracasei* or *Bifidobacterium lactis* for 12 weeks. Authors showed that probiotics did not provide any additional benefit to topical treatment of eczema and they also reported that probiotics did not prevent progression of allergic disease up to age 3 years.

The group of Rosenfeldt performed two trials [58,59], in 2003 and 2004, evaluating the effect of a mix of *Lactobacillus rhamnosus* and *reuteri* in children with moderate to severe AD. In these studies patients were randomized to receive placebo followed by active treatment or probiotic strains followed by placebo. Results of these trials showed a moderate improvement of clinical severity of eczema in children supplemented with mixture of probiotic strains with a more pronounced effect in patients with a positive skin prick test; moreover authors demonstrated that probiotic supplementation may stabilize intestinal barrier function with clinical benefit for children with AD. In 2005, Viljanen *et al*. [60] randomized 230 infants with AD and suspected cow’s milk allergy receiving *Lactobacillus GG*, a mix of probiotic strains (*LGG, L rhamnosus LC705, Bifidobacterium breve, Propionibacterium freudenreichii* ssp. *Shermanii JS*) or placebo for 4 weeks. A significant reduction in SCORAD score was recorded only in infants with IgE-associated AD in the LGG group, without any other significant beneficial effect between the probiotic supplementation *vs.* placebo. Similar results have been found by Sistek *et al*. [61] in 53 children with AD supplemented for 3 months with a mix of *Lactobacillus rhamnosus* and *Bifidobacterium lactis*. Results showed a decreasing in severity of AD only in a subgroup of children sensitized to food but not in children sensitized to environmental allergens. More recently, Gerasimov *et al*. [62] evaluated a 8-week supplementation of a mixture of *Lactobacillus acidophilus* and *Bifidobacterium lactis* in 90 children with moderate to severe AD, demonstrating a significant improvement in clinical severity in treated patients. Another recently published trial by Yeşilova *et al*. [63] evaluated a probiotic mix of *Bifidobacterium bifidum, Lactobacillus acidophilus, Lactobacillus casei*, and *Lactobacillus salivarius* in 40 children with moderate to severe AD. The 8-week intervention significantly reduced the SCORAD index in the treated children respect to placebo group. Moreover, probiotics supplementation significantly reduced the serum cytokines IL-5, IL-6, IFN-γ and total serum IgE levels.

Three other published studies were performed to evaluate the effect of probiotic strains in association with a prebiotic preparation. Passeron *et al*. [64] randomized 39 children with moderate to severe AD to assume *Lactobacillus rhamnosus Lcr35* plus a prebiotic or an identical appearing prebiotic preparation, finding that a synbiotic treatment was not superior to prebiotic alone. Analogous results were found by van der Aa *et al*. [65] in 2010: a synbiotic mixture (*Bifidobacterium breve* plus galacto-fructo-oligosaccharide) or placebo were randomly administered to 82 infants with AD. Authors concluded that synbiotic mixture does not have a beneficial effect on AD severity. On the contrary, very recently, Wu *et al*. [66] compared the effects of *Lactobacillus salivarius* plus fructo-oligosaccharide or prebiotic alone for 8 weeks on 54 children with moderate to severe AD. At 10 weeks, results of this trial showed a superior role of synbiotic combination for treating AD.

Despite the amount of data, reviews, meta-analysis and a Cochrane Collaboration review published in last 4 years do not definitively clarify the role of probiotics in treatment of PAD. The majority of these reviews [40,42,43,67,68] and the Cochrane Collaboration review [69] concluded that there is not enough evidence to support a recommendation for the use of probiotics for treatment of AD in children. Only Michail *et al*. [70] reported data from a meta-anlysis suggesting that there is a modest role for probiotics in treatment of moderately severe pediatric AD.

## 5. Conclusions

Analyzing data from the randomized controlled studies evaluated in this review, we can observe that a single strain of probiotic administration was studied more than mixtures of strains. Although we noted that studies performed with a mixture of probiotic strains show to be slightly more efficacious than single strain studies, there is currently insufficient evidence to suggest a mixture probiotic strains rather than a single strain supplementation. This hypothesis is in accordance with other authors [60] suggesting that a mixture of probiotic strains may be no better than a single probiotic. According to the literature, the most studied strains were Lactobacilli (especially LGG) and Bifidobacteria (especially Bb12), and the first one, both when used in single administration or in a mixture of strains, seems to be the most effective. Nevertheless, regardless of specific probiotic strain, a dose less than of 10^9^ cfu/day should not be administered.

However, taken together results are conflicting. The vast heterogeneity in study design, regarding for example the characterization of patients (number, age, severity of disease, sensitization to allergens, presence of other allergic diseases), dosage of probiotics, duration of supplementation, use of a single strain or a mixture and length of follow-up are possible explanations. Moreover, trials are complicated by natural tendency of AD to improve over time. So, before a recommendation to perform further large, well-designed randomized controlled trials may be done, clear guidelines to delineate the use of probiotic strains in clinical trials are needed. In 2010, a group of authors [71] proposed these guidelines in a review for study design, target populations, selection of placebo and probiotic microorganism(s), duration of follow-up, outcome and endpoint measurements, safety assessments and regulatory considerations. So, only with a significant reduction in heterogeneity of studies, results from future trials will be useful for patients.
